# Ipsilateral Cervicodeltopectoral Flap: A Forgotten Technique, Revival in the Era of Microvascular Reconstruction

**DOI:** 10.1007/s12663-022-01757-8

**Published:** 2022-07-08

**Authors:** Nawaz Usman, Punit Singh Dikhit, Naveena A. N. Kumar, Preethi S. Shetty, Keshava Rajan, V. N. R. Vittamsetti, Diksha Dinker, Anmi Jose

**Affiliations:** 1 Department of Surgical Oncology, Manipal Comprehensive Cancer Care Centre, Kasturba Medical College, Manipal Academy of Higher Education (MAHE), Manipal, Karnataka 576401 India; 2 Department of Pharmacy Practice, Manipal College of Pharmaceutical Sciences(MCOPS), Manipal Academy of Higher Education(MAHE), Manipal, 576104 India

**Keywords:** Head and neck reconstruction, Cervicodeltopectoral flap, CDP flap, Locoregional flaps, Pedicled flaps

## Abstract

Oral cavity cancer is one of the most common cancers in India responsible for significant morbidity and mortality in Indian subcontinent. Majority of cases present in advanced stages which requires extensive reconstruction following tumor resection. Microvascular free flap reconstruction is now considered standard of care for reconstruction for major head and neck skin-mucosal defects but, many factors still act as hindrance like patient’s comorbidities, long operating hours for microvascular reconstruction, logistic and financial issues from patient’s side. In such situation it is better to have a backup plan for reconstruction of major head and neck defects using pedicled flaps. Pectoralis major myocutaneous (PMMC) flap has been the workhorse flap for head and neck reconstruction since its introduction four decades ago. But relying too much on PMMC flap for major skin-mucosal defects especially in female patients is associated with complications and risk for flap failure leading to catastrophic and significant patient morbidities. Our study involves the use of two flaps for head and neck reconstuction involving skin-mucosal defects i.e PMMC flap for mucosal defect and cervicodeltopectoral (CDP) flap for skin defect. As of now there has been no retrospective or prospective study done which has given a conclusive statement regarding use of these two flaps simultaneously for head and neck reconstruction to the best of our knowledge. In our experience from the present study, CDP flap offers an excellent alternative for extensive head and neck reconstruction and can be readily included in the surgeon’s armamentarium with proper planning and meticulous handling.

## Introduction

Head and neck squamous cell carcinoma (HNSCC) is one of the commonest malignancies in the Asian subcontinent [1]. As per GLOBOCAN 2020, HNSCC is the third most common cancer in Indian population, especially in males. The major subsite of presentation for these patients is the oral cavity [1]. A majority of patients with Oral cavity squamous cell carcinoma (OCSCC) present in advanced stages [2], making simultaneous resection and reconstruction challenging. With the introduction of microvascular reconstruction techniques, this problem has been solved partly. Although an attractive option, microvascular expertise and tools are not freely available to the majority of the patients with OCSCC. The situation is complicated by the fact that the majority of patients with OCSCC in the Indian subcontinent belong to a lower socioeconomic status [2] and the added costs of microvascular surgery are often prohibitive.

This has led to locoregional flaps gaining widespread interest among Indian surgeons. Pectoralis Major myocutaneous flap (PMMC) has enjoyed the highest popularity amongst these locoregional reconstructive options. There have been many publications on the PMMC flaps, citing advantages and disadvantages of the same over the last two decades [3–6]. Although, it is a robust flap that covers large defects, situations which necessitates skin resection along with the primary tumour requires a modification that requires the flap to be bipaddled. This requires a larger paddle to be harvested from the chest, which can compromise flap viability, especially at the edges. Furthermore, harvesting larger flaps in females is a challenge. The inset of this flap is technically challenging as well. The Cervicodeltopectoral (CDP) flap is an attractive alternative in these situations. Easy to harvest and inset, the flap can be used to cover large skin defects without the necessity to bipaddle a PMMC flap. We here present our experience of reconstruction with CDP flap and PMMC flap in patients requiring combined reconstruction of skin-mucosal, cheek, and neck defects.

## Materials and Methods

The study was conducted in the Department of Surgical Oncology in Kasturba Medical College, Manipal. Clinical records of patients who underwent surgical resections for HNSCC followed by reconstruction with a combined ipsilateral CDP+PMMC flap between March 2019 and March 2020 were screened. Only those patients with a follow-up of 1 year were included in the study. Patients were evaluated for flap related complications like flap necrosis, wound infection, wound dehiscence, orocutaneous fistula and the status of the flap after one year.

## Technique of Flap Harvesting

**Fig. 1:**
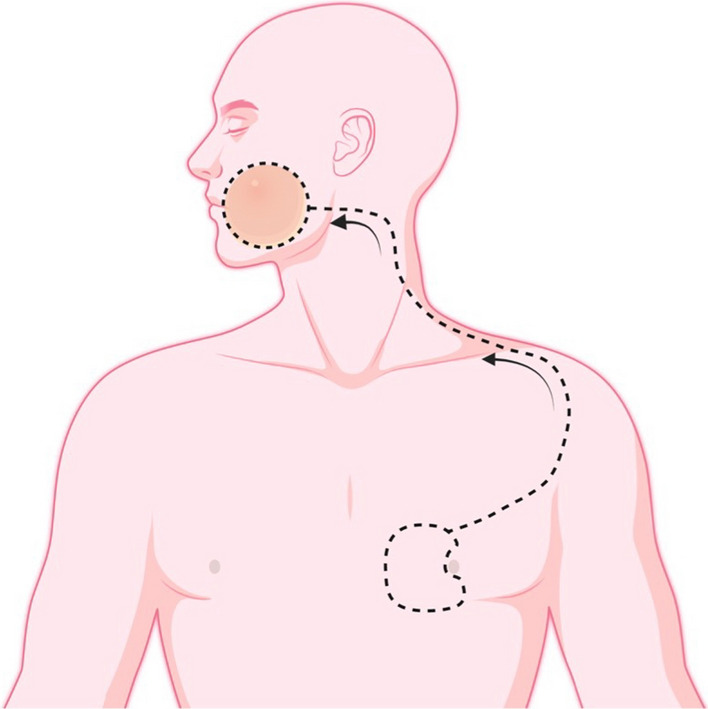
Flap marking indicating the planned primary skin defect along with outline for CDP flap and PMMC flap skin island medial to nipple. Arrow indicates planned rotation for CDP flap

**Fig. 2:**
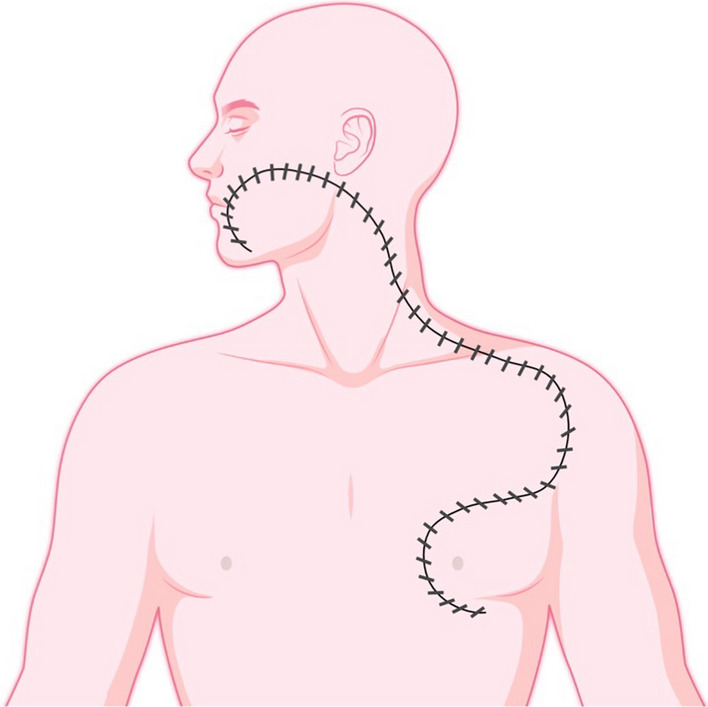
Final position of CDP flap after rotation thus closing the primary defect and final suture line

**Fig. 3:**
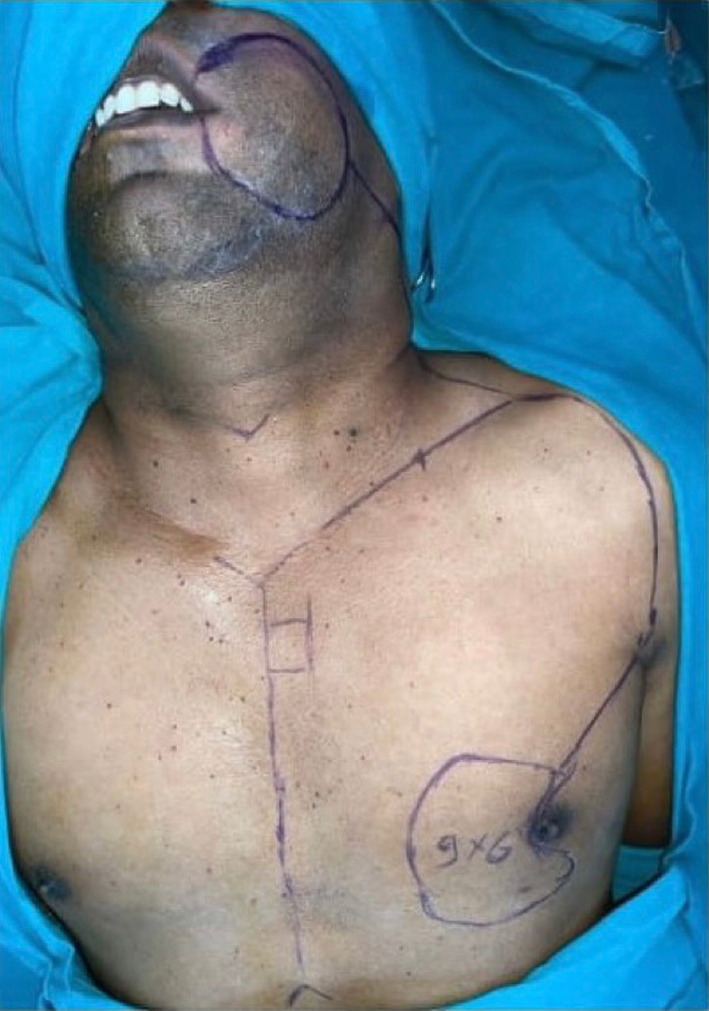
Patient with OCSCC with planned surgical resection including extraoral cheek skin and CDP flap markings

**Fig. 4:**
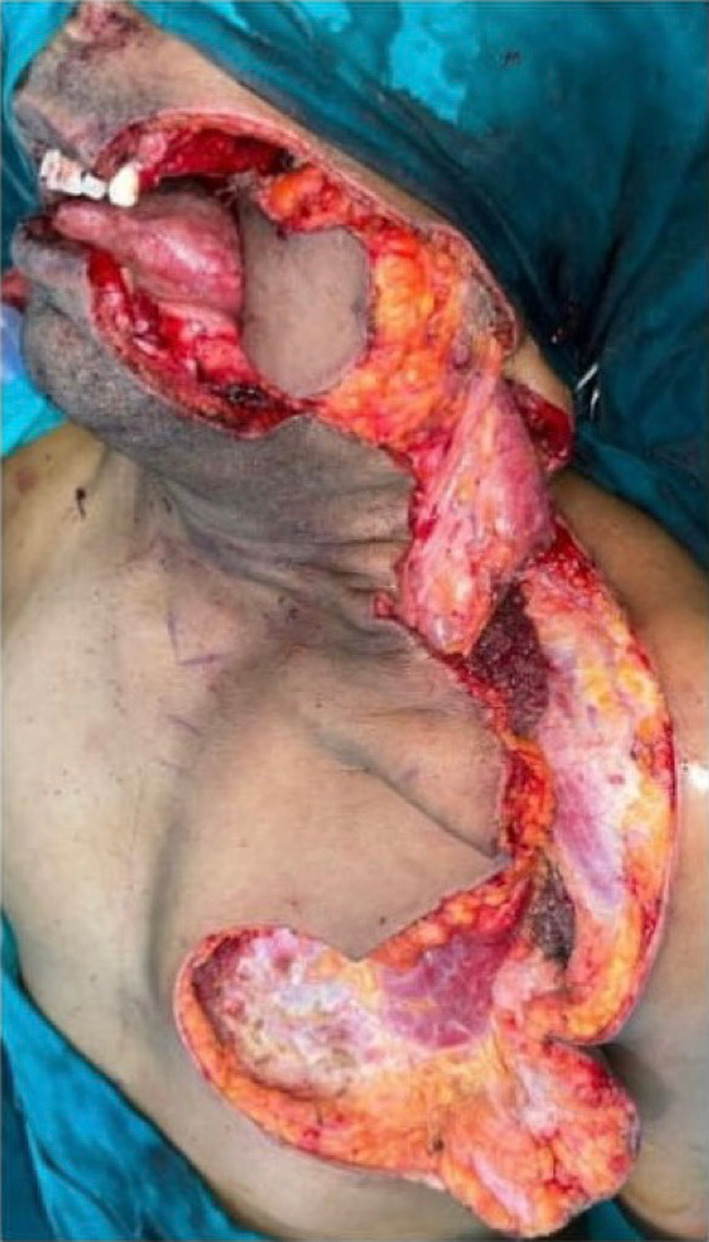
Post tumour resection defect, CDP flap raised and rotated with PMMC flap in position within oral cavity

**Fig. 5:**
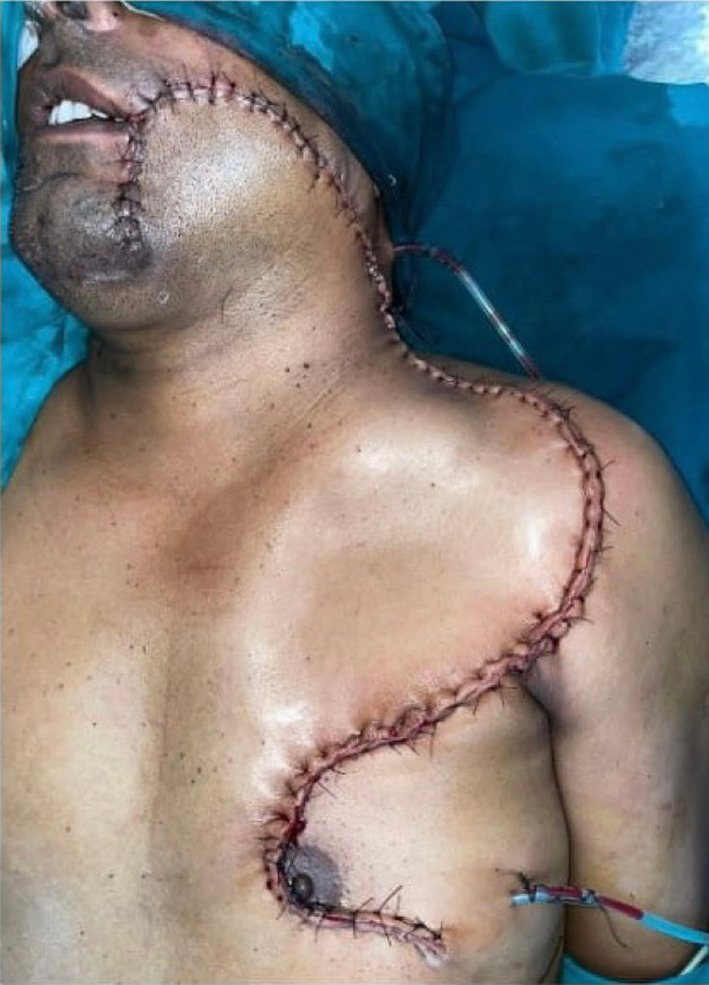
Final flap position after closure

Flap marking is done in the Rose position before starting the procedure. The marking is done in such a way that the superior border of flap rotates towards the inferior margin of the defect (Figs. 1 and 2). The flap marking extends from the latero-inferior border of the defect, following the earlobe up to the mastoid process, and then is extended down along the neck, 2 cm behind the anterior border of the trapezius muscle. It is then extended across the acromio-clavicular joint along the lateral border of the pectoralis major muscle. This incision is then connected to the release incision of the planned PMMC flap, from the superolateral border of skin paddle (Fig. 2a). The flap is then elevated in the subplatysmal plane in the neck, and under the pectoralis fascia in the chest [7–10]. The anterior extent is the border of sternohyoid muscle, which forms the anterior limit of neck dissection. Once flap markings are delineated for the CDP flap, the PMMC flap with skin paddle is planned medial to the nipple, well within the extensions of pectoralis major, with release from superolateral aspect of skin paddle towards the axilla (Fig. 2b). The PMMC flap is then harvested, preserving the internal mammary perforators which supply the CDP flap. Once the PMMC flap raising is complete, it is transferred over the clavicle to cover the mucosal defect, while CDP flap is rotated to cover the skin defect (Figs. 3, 4 and 5).

## Result

Records of 30 patients operated for OCSCC who underwent reconstruction using the CDP flap and PMMC flap simultaneously between March 2019 and March 2020 were screened. Only those patients who completed the follow-up period of 1 year were included in the study. A total of 25 patients were included in the study. All of these patients underwent reconstruction with a CDP flap for skin defects along with a PMMC flap for intraoral mucosal defects. All the patients included in the study underwent adjuvant therapy as per the final histopathology reports after a tumour board discussion. The details of patient demographics, primary site, and defect size as per the surgeon’s notes, adjuvant therapy and associated complications are mentioned in Table 1. There were three flap related complications, with one patient developing OCF on postoperative day four. The other two patients developed marginal flap edge necrosis at the superior most edge of the flap- recipient skin margin, which were managed conservatively with debridement and resuturing.

**Table 1:** Details of patient included in the study

S. No	Age/Sex/Morbidity	Primary site	Skin defect	Adjuvant therapy	Complications
1 2 3 4 5 6 7 8 9 10 11 12 13 14 15 16 17 18 19 20 21 22 23 24 25	54/M/DM 48/F/HTN 62/F//Nil 52/F/Nil 68/M/Nil 72/M/Nil 68/F/Nil 53/F/Nil 56/F/HTN 59/F/HTN/DM 68/M/HTN 70/M/Nil 76/F/DM 61/M/Nil 48/M/Nil 57/F/Nil 56/M/Nil 61/M/Nil 66/F/Nil 70/M/Nil 71/F/Nil 51/M/Nil 62/F/HTN 63/F/HTN 66/M/Nil	Lower GBS Lower GBS Lower alveolus + tongue Lower alveolus + tongue RMT Lower alveolus + tongue Lower alveolus + tongue RMT Buccal mucosa Upper Alveolus RMT Lower alveolus + tongue Lower alveolus + tongue Lower alveolus + tongue Lower GBS Upper alveolus + RMT RMT Lower GBS Lower GBS Upper alveolus + RMT Tongue + lower alveolus RMT Lower alveolus + GBS Lower GBS Lower alveolus	IO-7*8, EO-5*6 cm IO-7*7, EO-5*4 cm IO-5*6, EO-6*6 cm IO-7*7, EO-5*5 cm IO-6*6, EO-4*3 cm IO-6*5, EO-4*4 cm IO-5*5, EO-4*3 cm IO-5*6, EO-4*4 cm IO-7*5, EO-4*5 cm IO-6*5, EO-3*3 cm IO-4*4, EO-4*6 cm IO-5*6, EO-6*3 cm IO-7*7, EO-5*3 cm IO-5*6, EO-4*3 cm IO-7*5, EO-5*4 cm IO-7*6, EO-3*4 cm IO-4*4, EO-2*3 cm IO-4*5, EO-5*4 cm IO-7*6, EO-3*4 cm IO-7*6, EO-5*4 cm IO-7*7, EO-4*4 cm IO-4*5, EO-3*4 cm IO-7*9, EO-4*3 cm IO-5*4, EO-4*3 cm IO-4*5, EO-2*3 cm	RT RT RT RT RT CT-RT RT RT CT-RT RT RT RT CT-RT RT RT CT-RT RT CT-RT RT CT-RT CT-RT RT CT-RT RT RT	Marginal necrosis Nil Nil Nil Nil Nil Nil Nil Nil Nil Nil Nil Orocutaneous fistula Nil Nil Marginal necrosis Nil Nil Nil Nil Nil Nil Nil Nil Nil

*IO* Intraoral, *EO* Extraoral, *CT* Chemotherapy, *RT* Radiotherapy, *GBS* Gingivobuccal sulcus

## Discussion

Reconstruction following head and neck cancer resections have evolved drastically over the last few decades. Multiple options are available for reconstruction, like local flaps, regional flaps, and microvascular free flaps. Free flaps have paved the way for reconstruction of large defects with minimal donor site morbidity and maximal defect coverage. But there are a few inherent problems with microvascular reconstruction, including the lack of availability of expertise at every centre, logistic and financial issues, the patient’s general status and medical comorbidities, which restrict the use of free flaps [11]. These issues have necessitated surgeons to be familiar with locoregional reconstructive options. The PMMC flap has been the most popular amongst the locoregional reconstructions since its introduction over four decades ago.

Literature review has been suggestive of complication rates of around 10–50%, which includes infections, flap dehiscence, orocutaneous fistula (OCF), and marginal flap necrosis [3–5]. Bipaddle PMMC flap has been associated with major flap related morbidity, chiefly partial flap necrosis requiring secondary debridement. Two such case series reported flap necrosis with bipaddle PMMC in the range of 3–18% [3, 6]. Other complications associated and reported with bipaddle PMMC include flap dehiscence, infection, and orocutaneous fistula (OCF). Most of these OCF develop in primary tumours involving the tongue-floor of mouth complex, the proposed reason being the significant amount of dead space in the region following surgical resection [12].

Bakamjian first described the deltopectoral flap in 1965, the pedicle of which was based medially on internal mammary perforators [11]. After a decade, Daniel and colleagues defined the primary vascular zones of deltopectoral flap [7]. Later on, Becker gave a modification in which he included the cervical skin along with deltopectoral flap for reconstruction of large head and neck defects, as he was able to preserve the internal mammary perforators along with the supply from platysma muscle and base of neck [7]. The various cited advantages of the CDP flap are excellent colour and texture match with availability of adequate tissue adjacent to the primary defect and ability to perform neck dissection in same incision [11].

In our case series, during the period of one year, 30 patients underwent CDP flap reconstruction, out of which 25 patients were included in the study, keeping in view the inclusion criteria. All our patients underwent reconstruction with a double flap i.e. PMMC flap and CDP flap. None of our patients had total loss of either of the two flaps. The mean defect size in our case series was 34.56 and 16.52 cm^2^ for intraoral and extraoral defects, respectively. In the present case series, we noted three flap related complications, i.e. one OCF along with two minor flap edge necrosis. OCF was managed conservatively with strict maintenance of oral hygiene, saline flush through the fistula to prevent salivary-debris pooling, and by continuing naso-gastric feeding for three weeks. We modified our technique of CDP flap design as compared to CDP design by other authors [7–10] by connecting the incision from the neck over the acromio-clavicular joint to the release incision of the skin paddle of the PMMC flap over the superolateral border. By this connection, we completely avoided the incision on the skin paddle medial to the nipple, thus preserving the skin for the planned PMMC flap. This modification makes sure that PMMC skin paddle is situated at the most vascular zone, while at the same time preserving the axis of rotation of the CDP flap without over torquing the internal mammary perforators thus, minimising the risk of flap necrosis for either of the two flap units.

The idea of using the PMMC flap as a sole unit for reconstruction in females has always been a matter of concern amongst surgeons. The situation is even more challenging when there is a combined cheek and mucosal defect, which requires a bipaddle PMMC flap reconstruction. In a study of 168 patients undergoing PMMC flap reconstruction by Kroll et al., a major finding was that flap failure rates were significantly higher in female patients as compared to male patients [13]. Similar findings were echoed in the study by Chakrabarti S et al., with a higher rate of total flap loss as compared to males [14]. The reason behind this disparity is the redundancy of the breast tissue in females, predisposing the cutaneous perforators to inadvertent injury during flap handling. So, a through and through cheek-mucosal defect in female patients stretches the PMMC flap to its threshold for defect coverage, predisposing it to major flap related complications.

For defects involving combined tongue-alveolus, or tongue -floor of mouth complex with a skin defect, the dual reconstruction plan of CDP and PMMC flap is a better option in our opinion. The peculiar thing about this anatomical site of primary tumour location is that the floor of the mouth has a non-keratinised extra thin mucosa, and after adequate resection following oncological principles, the remaining mucosa is unforgiving if the closure is under tension. This site is also prone for salivary pooling and is constantly exposed to micromovements, starting from the postoperative period when the patient attempts to speak, which can result in flap margin dehiscence and finally OCF. Thiagarajan et al. also shared the same experience in their study [12]. In our study, there were eight patients with the primary tumour involving tongue-alveolus-floor of mouth complex, out of which five were females. One of these female patients developed an OCF on postoperative day four, which was managed conservatively without any delay in planned adjuvant treatment. None of the other patients developed any major flap related complications despite having high risk features for OCF. We managed to minimise the complications by reducing the burden on a single flap, i.e. on the PMMC flap for intraoral coverage. Since the extraoral defect was already planned to be reconstructed with a CDP flap, there was no compulsion to rotate the PMMC flap for bipaddling, thereby avoiding putting the cutaneous perforators at risk. Thus, the bulk of PMMC flap could be placed at the OCF susceptible site that is near the tongue-floor of mouth region, so as to provide tensionless watertight closure, minimising the risk.

Our experience with the present study and ongoing cases is that combining the PMMC flap with a CDP flap is a better reconstructive option, rather than relying on a single flap unit for such complex scenarios. In the present case series, the largest skin defect that could be covered using a CDP flap was about 6 * 6 cm with a  major complication rate of 4% without any incidence of total or partial flap necrosis. The other advantage of the CDP flap is that there is no requirement for a separate incision for neck dissection, as it can be carried out from the already planned incision with excellent exposure. Another major utility of the CDP flap is its excellent donor-recipient site colour match, which makes it better suited for even reconstruction of commissure defects, which becomes a tedious job if only a PMMC flap or primary closure is planned. In our study, two patients had a lip commissure defect which was reconstructed using adequate release and rotation of the CDP flap with minimal microstomia postoperatively.

The advantage of the CDP flap, as per our experience, is that it can be modified according to the defect size and shape without much difficulty. Recently, we have described our technique of contralaterally based CDP flap for coverage of head and neck defects [11]. The CDP is a versatile flap with many applications. The technique of flap elevation and defect coverage is simple, reliable, robust and can be easily used in resource constrained situations, even in salvage cases post chemoradiation.

## Conclusion

The CDP flap is an old technique of head and neck reconstruction, but its utility has been greatly restricted in the era of microvascular reconstruction. The CDP can be a simple, reliable flap for reconstruction of skin defect along with other local flaps without any major complications, particularly in resource limited settings.
